# Serum extracellular vesicles 3’tRF-ThrCGTand 3’tRF-mtlleGAT combined with tumor markers can serve as minimally invasive diagnostic predictors for colorectal cancer

**DOI:** 10.3389/fonc.2024.1474095

**Published:** 2024-10-21

**Authors:** Jiefei Peng, Fan Bu, Lei Duan, Anna Song, Guojun Wang, Zhijun Zhang

**Affiliations:** ^1^ Department of Clinical Laboratory, Affiliated Taian City Central Hospital of Qingdao University, Taian, China; ^2^ Shandong Provincial Key Medical and Health Laboratory of Anti-Drug Resistant Drug Research, Affiliated Taian City Central Hospital of Qingdao University, Taian, China; ^3^ Department of Reproduction and Genetics, Affiliated Taian City Central Hospital of Qingdao University, Taian, China; ^4^ Department of Neurosurgery, Affiliated Taian City Central Hospital of Qingdao University, Taian, China

**Keywords:** colorectal cancer, serum EV, 3’tRF-ThrCGT, 3’tRF-mtlleGAT, minimally invasive diagnostic predictors, tumor markers

## Abstract

**Background:**

Colorectal cancer (CRC) is a leading cause of morbidity and mortality, and timely diagnosis and intervention are crucial for cancer patients. Transfer RNA-derived fragments (tRFs) play a noncoding regulatory role in organisms. Serum EV(extracellular vesicles), as an integral mediator of intercellular transmission of genetic information vesicles in Transfer RNA-derived fragment (tRF RNA), are expected to be minimally invasive diagnostic and predictive biologic factors of CRC.

**Methods:**

Collect serum samples from 205 CRC patients, and then isolate extracellular vesicles from the serum. Captured the physical morphology of EV through transmission electron microscopy. The particle size was detected by particle size assay, and protein expression on the surface of EV was verified by Western blot. Gene microarrays were screened for differentially expressed tRF-RNA. TRF RNAs were verified by qPCR for differential expression in 205 CRC patients and 201 healthy donors, assessing the CRC diagnostic efficiency by area under the curve (AUC).

**Results:**

Compared with 201 healthy donors, CRC patients experienced significantly down-regulated serum EV 3’tRF-ThrCGT while significantly up-regulated 3’tRF-mtlleGAT. Serum EV 3’tRF-ThrCGT and 3’tRF-mtlleGAT predictive diagnostic efficiency: 0.669 and 0.656, and the combination of CEA and CA724 predictive diagnostic efficiency was 0.938.

**Conclusion:**

The study data showed that 3’tRF-ThrCGT and 3’tRF-mtlelGAT can be minimally invasive diagnostic CRC indicators. The combination of tumor markers CEA and CA724 has important diagnostic significance.

## Background

Despite the gradual improvement in living standards, the incidence of colorectal cancer (CRC) remains high, ranking third globally and having the second-highest fatality rate among all cancers. Colorectal health is closely related to overall quality of life (1). CRC incidence is significantly higher in urban areas than rural areas, and there is a strong correlation with dietary structure, lifestyle, and socioeconomic status (2, 3). A multitude of factors contributes significantly to CRC development, particularly age and hereditary factors. Hereditary CRC syndromes: Lynch syndrome (hereditary nonpolyposis CRC), familial adenomatous polyposis, and MUTYH-associated polyposis are strongly associated with CRC development (4–6). Exploring early diagnostic modalities for CRC may significantly reduce mortality and alleviate suffering in cancer patients.

TRF RNAs, also known as tRNA-derived small RNAs (TDRs), are fragments of precursor or mature tRNAs with different lengths and sizes. TRF RNAs are a recently discovered group of short noncoding RNAs (ncRNAs) that are produced enzymatically from tRNAs and lengthened 14–50 nucleotides (NT). Noncoding RNAs, including this particular example, have significant regulatory functions in organisms. Based on their biogenesis location, TDRs are typically classified into tRNA half-fragments and tRNA-derived short RNA fragments (tRFs) (7, 8). The biogenesis of tRF RNA is a degradation product of tRNAs, the production of which is not randomly spliced but is actually governed by highly conserved and precise site-specific cleavage mechanisms (9). Depending on the cutting position, it is categorized into 5’tRFs, 3’tRFs, and intermediate tRFs (10–12). TRF RNAs are crucial in gene transcription and post-transcriptional expression, as well as cellular metabolism (13).

EV, as an important communication medium, contain many genetic information. Many ncRNAs are involved in gene regulation and regulating the expression of downstream genes, such as tRF RNAs. By extracting the contents of EV (14, 15), the aim was to infer the important role of gene expression regulation. Current research has revealed that EV exist in multiple forms: blood, breast milk, urine, and various secretory fluids of the human body. Studies have shown that cells help transmit genetic information via tRF RNA carried by EV (16, 17). They also carry medications for therapeutic purposes to be developed (18, 19). EV are frequently utilized in innovative clinical diagnostics to monitor malignancy occurrence and the degree of disease progression promptly. This offers a highly dependable foundation for prompt cancer detection and management (20–22).

To monitor the information transfer between CRC cells by understanding tRF RNA differential expression in serum EV and then to determine the clinicopathologic relationship with CRC patients. Minimally invasive diagnosis and early interventional treatment of CRC provide a basis for reducing CRC patients’ mortality (23). The integration of tumor markers with tRF RNAs compensates for the shortcomings of traditional CRC diagnosis that rely on colorectoscopy, such as the high cost of the test, invasiveness, long duration, and low patient acceptance. We developed a minimally invasive diagnostic modality combined with tumor markers for comprehensive screening and timely monitoring of cancer incidence.

## Materials and methods

### Specimen preparation, clinicopathologic analysis and study design

All the blood samples of CRC patients were before treatment, and the specimens were collected on the same day when the patients finished collecting the test. The collection of serum specimens was between March 2021 and April 2022; we collected 205 samples from CRC patients and 201 samples from healthy donors (screened from a population of healthy physical examination) from The Affiliated Taian City Central Hospital of Qingdao University. The selected sample type is 3mL of coagulation promoting whole blood containing separation gel and centrifuged at 4°C for 1000g for 10 minutes. We collected the supernatant into a 1.5ml EP tube and stored it in a -80°C refrigerator. The case details of colorectal patients were reviewed: gender, age, cancer type, patient’s lifestyle habits, and tumor metastasis.

In the training stage, these 20 dysregulated Ex-tRF RNAs were determined by qRT-PCR using 48 HDs and 48 CRC samples. In the testing stage, the identified Ex-tRF RNAs were validated in serum EV samples of 72 HDs and 72 CRC patients. In the last stage, 166 samples (81 Health donors and 85 CRCs) were collected to further evaluate the Ex-tRF RNAs in CRC.

### Extraction and analyses of extracellular vesicles

All collected serum samples 1.5mL were thawed on ice and centrifuged at 10,000g for 30 min at 4°C. The supernatant was then centrifuged at 100,000g for 2 hours at 4°C using a Type 50.4 TiRotor (Beckman Coulter) to isolate the EV. The extraction methods followed those described by Zhang ZJ, Xie L et al. (23, 24). Extracted EV size and distribution were determined through a particle size potentiostat qNano (New Zealand) The parameters set for measuring particle size in qNano include a size determination accuracy of 1nm, a concentration range of 10 ^ ^5^-10 ^ ^13^ particles/mL, and a sample size of 40μL. The EV were placed on a copper grid with a 50 µl drop of 1% glutaraldehyde, and transferred to 100 µl distilled water after 5 min. The grids were left undisturbed for 2 min and stained with 50 µl oxalyl uranyl solution (pH 7) for 5 min. The grids were rinsed 7 times with distilled water for 2 min. We observed their physical morphology using a transmission microscope FEI Tecnai T20e (FEI Company, USA).

Western blotting was performed for the identification of EV proteins on PVDF membranes (Bio-Rad, America, 1620177). Reference (25) was consulted to obtain EV surface high expression of proteins TSG101(Proteintech, America, 14497-1-AP), CD63(Proteintech, America, 25682-1-AP) and CD81(Proteintech, America, 66866-1-Ig). The secondary antibody followed a 1h incubation at 37°C, using an ECL reagent (Bio-Rad, America, 1705060) to visualize the bands.

### RNA extraction and reverse transcription

The extracted EV were resuspended with 1 mL of PBS (Solarbio, China, P1022-500ml), adding 1 mL of Trizol (Invitrogen, America, 10296010-100ml) to fully lyse the EV to extract RNA, reversely transcribing the extracted RNA into cDNA (AG, China, 11701) for the subsequent qPCR (AG, China, 11717). The experimental procedure was susceptible to RNA degradation during operation; all the procedures were always performed on ice.

### Small RNA microarray analysis

Herein, we sampled EV from three groups of healthy controls and six groups of CRC patients by microarray (Arraystar Small RNA Expression Chip, Kangcheng Biotechnology, China). In total, 22.5μL 2X Hybridization buffer (Agilent) was combined with the completed labeling process, resulting in a 45μL final volume. The mixture was subjected to heat of 100°C for 5 min and subsequently rapidly cooled to 0°C. The labeled sample mix, measuring 45 μL, was subjected to hybridization on a microarray at 55°C for 20 h. The slides were immersed in a solution of 6X SSC with 0.005% Triton X-102 at room temperature for 10 min, followed by a solution of 0.1X SSC with 0.005% Triton X-102 for 5 min. The slide scan was conducted using an Agilent G2505C microarray scan, analyzing the acquired array images via Agilent Feature Extraction software (version 11.0.1.1). The raw intensities were subjected to log2 transformation and quantile normalization. Following normalization, we only retained the probe signals that had Present (P) or Marginal (M) QC flags in at least three out of nine samples. Differentially expressed small RNAs were compiled by fold change and P-value cutoffs and annotated with genomic and biological info, scatter plots, volcano plots, and hierarchical clustering heatmap analyses. The FDR correction algorithm for P-value is the Benjamini-Hochberg method, and the computation is done in the programming language (R).

### qPCR and statistical analysis

The qPCR was performed to detect differentially expressed tRF RNA in healthy donors and CRC patients. The internal reference was U6, and we made statistical analyses based on ΔCT values (ΔCT=CT_CRC_-CT_U6_) (23, 26). The reagents used are SYBR Green (AG, China). Each data set was repeated independently three times (P<0.001). Statistical analyses were conducted through GraphPad Prism 6.0 (CA, USA) and SPSS 22.0 (Ehningen, Germany). The normally distributed numeric variables were analyzed by t-test, whereas non-normally distributed variables were evaluated by Mann–Whitney test. Analyzed the statistical significance of health donors and CRC patients. P < 0.05 indicated statistically significant. The diagnostic efficiency was evaluated using the receiver operating characteristic curve (ROC), represented by AUC curve.

## Result

### Serum EV identification

**Figure 1:**
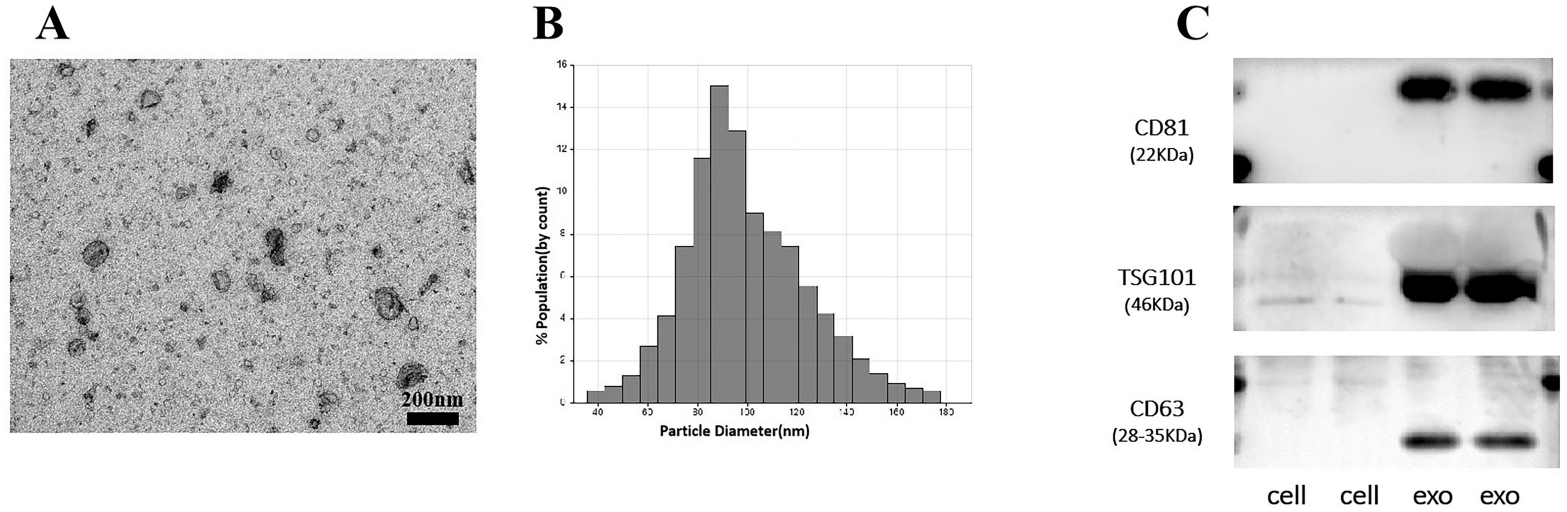
Performance verification of serum exosomes. **(A)** Identification of the physical morphology of exosomal by transmission electron microscopy (TEM). **(B)** Measurement of exosomal size distribution using particle size analyzer by qNano. **(C)** Western blot validated of serum exosomal surface protein markers.

Herein, we used ultracentrifugation to extract EV, which are vesicles that are the basis for the study of tumor metastasis and information transfer. We characterized and tested the properties of EV. The morphology of EV showed a rounded spherical shape, with a diameter distribution between 80–130 nm and a normal distribution of diameter size, mainly concentrated around 100 nm (**Figures 1A, B**). Western blot experiments verified the surface protein expression of EV vesicles, which was significantly different from the cell surface protein expression. The surface of EV highly expressed TSG101, CD81/63 proteins (**Figure 1C**). The above results showed that we successfully extracted EV. The clinical data of the patients were collected for pathological analysis, and 3’tRF-ThrCGT and 3’tRF-mtlleGAT did not statistically significantly differ in the patients’ clinicopathological information in terms of gender, age, life habits, pathology type, and tumor metastasis.

### Microarray of serum extracellular vesicles tRF RNA expression

**Figure 2:**
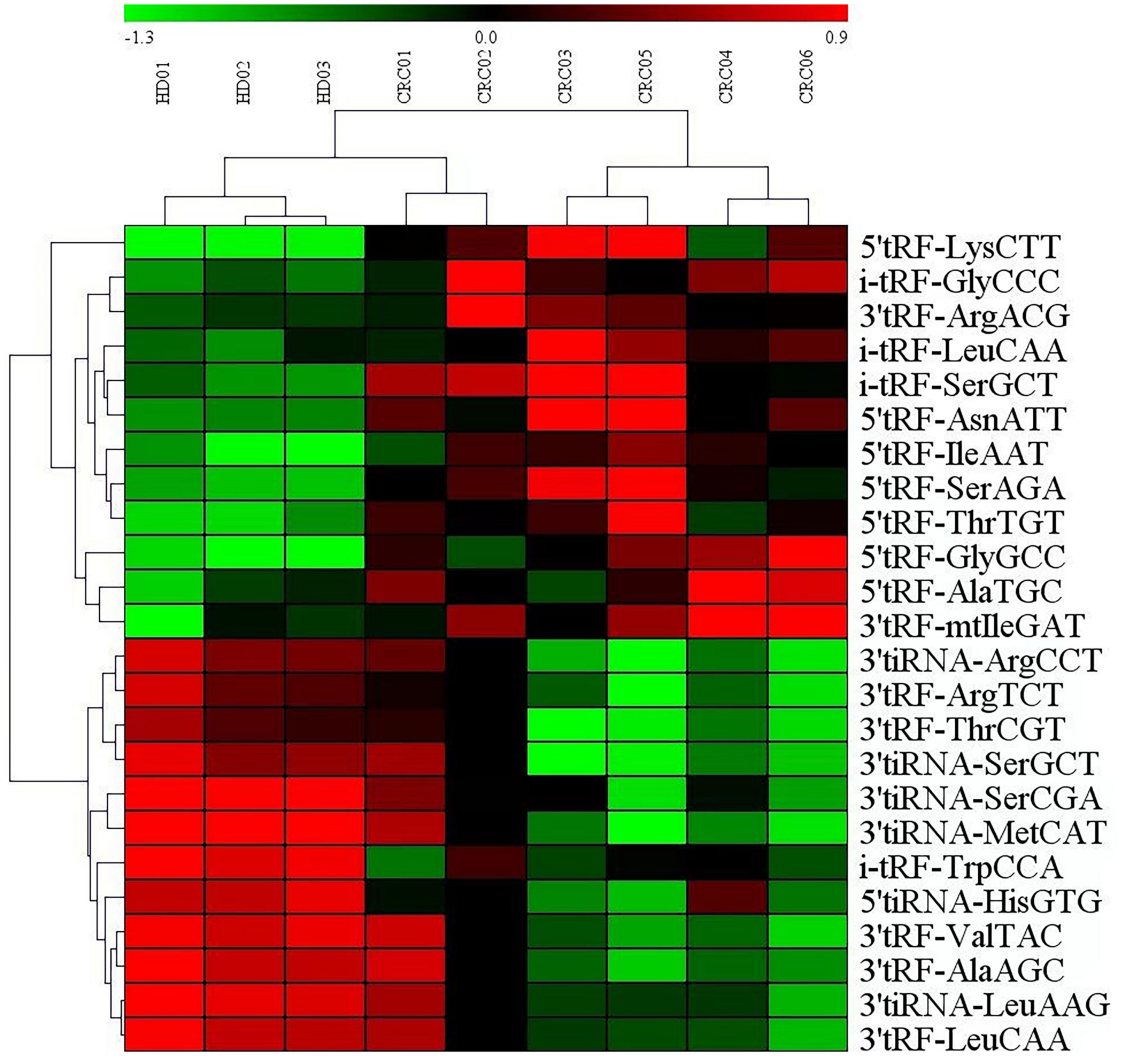
Exosomal miRNAs profile of the CRC patients. Cluster analysis of differentially expressed serum exosomal tRFRNAs between 6 CRC patients and 3 healthy donor.

Analysis of serum microarray results from CRC patients and normal controls revealed that 12 tRF RNAs were up-regulated and 12 tRF RNAs were downregulated (**Table 1**). **Figure 2** presents an analysis of the following three groups: healthy controls, nonmetastatic CRC patients, and metastatic CRC patients. The clustering diagram results show differentially expressed tRF RNAs. In the subsequent experiments, we validated tRF RNA expression in the serum of 205 cases of CRC through quantitative PCR analysis. The microarray data in this study has been stored in genome sequence archives (Genomics, proteomics and bioinformatics) Bioinformatics/Beijing Institute of Genomics, Chinese Academy of Sciencesaccessible to the public https://ngdc.cncb.ac.cn/omix/preview/KYegl8Sk (OMIX007492).

**Table 1:** tRFRNA expression profiling (12 up-regulated and 12down-regulated).

tRFRNA	Fold change	Description	P value	tRFRNA	Fold change	Description	P value
5'tRF-LySCTT	5.2820	Up	0.0352*	3'tiRNA-ArgCCT	0.4563	Down	0.0252*
i-tRF-GlyCCC	1.9494	Up	0.0040**	3'tRF-ArgTCT	0.4862	Down	0.0091**
3'tRF-ArgACG	1.5124	Up	0.0406*	3'tRF-ThrCGT	0.4630	Down	0.0436*
i-tRF-LeuCAA	1.6801	Up	0.0298*	3'tiRNA-SerGCT	0.4095	Down	0.0407*
i-tRF-SerGCT	2.4099	Up	0.0068**	3'tiRNA-SerCGA	0.3619	Down	0.0048**
5'tRF-ASnATT	2.4070	Up	0.0192*	3'tiRNA-MetCAT	0.2757	Down	0.0075**
5'tRF-IleAAT	2.3299	Up	0.0062**	i-tRF-TrpCCA	0.4491	Down	0.0007***
5'tRF-SerAGA	2.4687	Up	0.0018**	5'tiRNA-HisGTG	0.4754	Down	0.0080**
5'tRF-ThrTGT	2.2617	Up	0.0040**	3'tRF-ValTAC	0.4447	Down	0.0148*
5'tRF-GlyGCC	3.2835	Up	0.0009***	3'tRF-AlaAGC	0.4406	Down	0.0104*
5'tRF-AlaTGC	1.8001	Up	0.0435*	3'tiRNA-LeuAAG	0.4697	Down	0.0081**
3'tRF-mtleGAT	2.5840	Up	0.0015**	3'tRF-LeuCAA	0.4978	Down	0.0321*

*P<0.05; **P<0.01; ***P<0.001. The FDR correction algorithm for P-value is the Benjamini-Hochberg method, and the computation is done in the programming language (R).

### Serum extracellular vesicles 3’tRF-ThrCGTand 3’tRF-mtlleGAT for diagnosis of CRC and clinicopathologic associations

**Figure 3:**
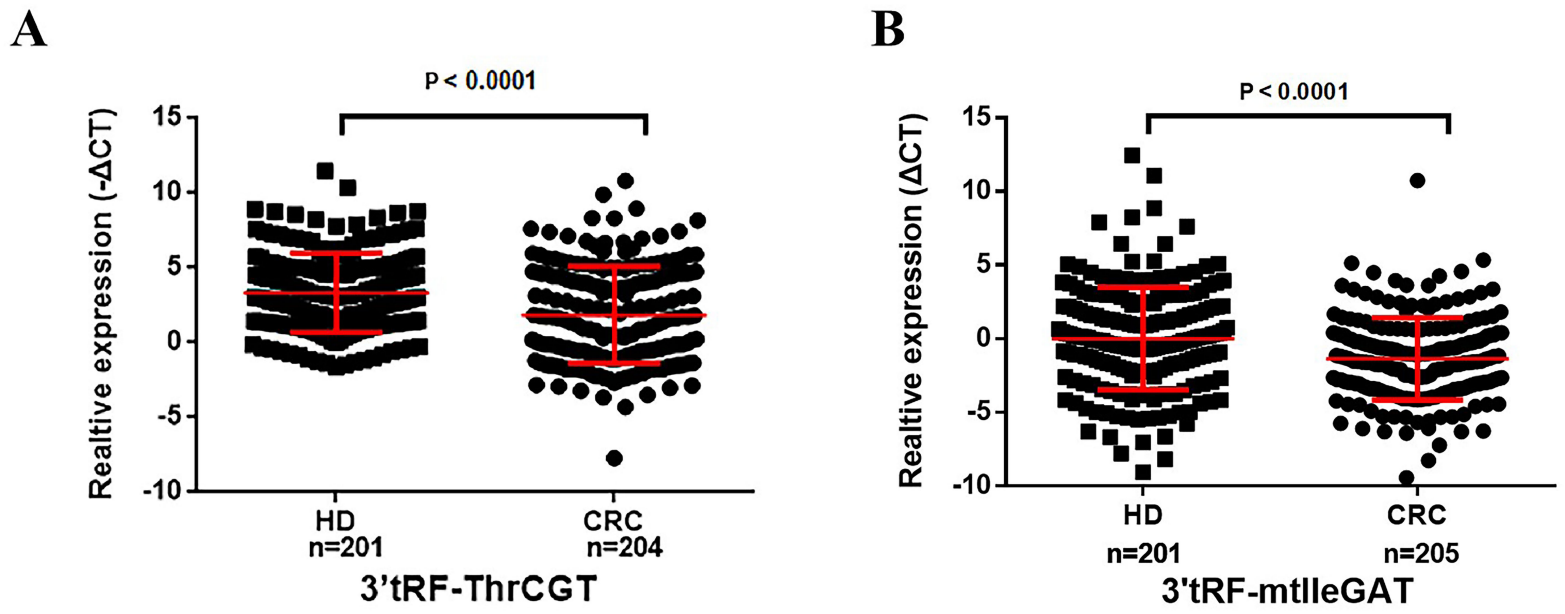
Serum exosomal 3’tRF-ThrCGT and 3’tRF-mtlleGAT are potential biomarkers of colorectal cancer. The expression levels of serum exosomes 3’RF-ThrCGT **(A)** and 3’tRF-mtlleGAT **(B)** in CRC and healthy donors.

We have collected serum EV for reverse transcription and real-time quantitative PCR (ΔCT=CT_CRC_-CT_U6_) to explore the expression of serum EV 3’tRF-ThrCGTand 3’tRF-mtlleGAT in 205 colorectal cancer patients and 201 normal healthy donors. We verified the non-specific nature of the primers, and the melting curve showed a single peak with good specificity. The results are shown in **Supplementary Data Sheet 1**. The results of quantitative PCR elucidated 3’tRF-ThrCGT significant down-regulation while 3’tRF-mtlleGAT significant overexpression in the sera of cancer patients (**Figures 3A, B**). In order to display the research data more intuitively, we used the -ΔCT=-(CT_CRC_-CT_U6_) formula calculations for 3’tRF-ThrCGT. The data we obtained through quantitative PCR is consistent with the data from the microarray in **Figure 2**.

### Relationship of 3’tRF-ThrCGT and 3’tRF-mtlleGAT to clinicopathologic grading

**Figure 4:**
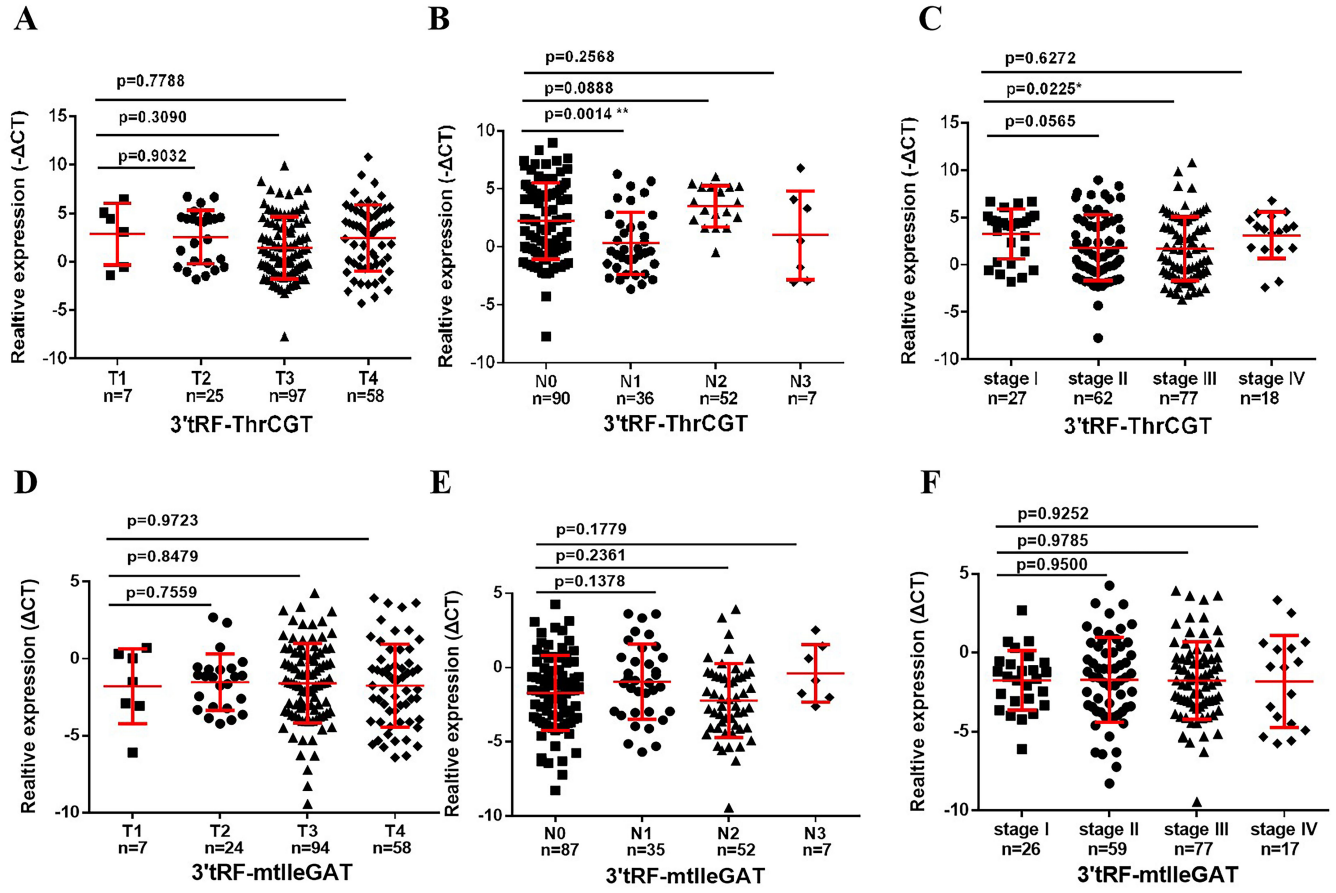
Association between the serum exosomal tRFRNA and tumor stage. Expression levels of 3’tRF-ThrCGT **(A)** and **(B)** in T and N stages patients. **(C)** Serum exosomal 3’tRF-ThrCGT expression in early stage (I+II) and advanced stage (III+IV) patients. The expression level is represented using -ΔCT. **(D, E)** Statistical levels of 3’tRF-mtlleGAT in T and N stages patients **(F)** Serum exosomal 3’tRF-mtlleGAT expression in stage (**P < 0.01, *P < 0.05, ns, not significant).

To ascertain the correlation between patients’ survival and tumor stage grading of CRC, early diagnosis of CRC occurrence has an important role in patients’ survival improvement. We screened that 3’tRF-ThrCGT was not statistically significant with T-stage grading of CRC primary foci (**Figure 4A**). CRC tumor metastasis is more pronounced in advanced stages, and tumor findings are generally more frequent in these patients compared to those in earlier stages. Clinical data show that lymph node metastasis (LNM) on N0/N1 was statistically significant (P <0.05) (**Figure 4B**). The number of patients with LNM found at the initial stage was higher compared to those without LNM. In the staging of the tumor stage I/III, there was statistical significance *P<0.05 (**Figure 4C**). The results showcased no statistical significance between stages I, II, and IV. 3’tRF-mtlleGAT was not statistically significant in the analytical data of the clinical staging of CRC patients in comparison with the tumor of the primary location. Stage T (**Figure 4D**), LNM (**Figure 4E**), and the clinical stage of the tumor (**Figure 4F**) did not significantly differ. Reviewing the lifestyle habits and case information of CRC patients revealed no statistically significant differences (**Table 2**).

**Table 2:** Characteristics of CRC patients for differentially expressed exosomes 3’tRF-ThrCGT and 3’tRF-mtlleGAT.

Characteristic		No. cases	3’tRF-ThrCGT		No. cases	3’tRF-mtlleGAT	
			Median with interquartile range	P-value		Median with interquartile range	P-value
Age (year)	≤62	73	-1.798 (-2.1909~-1.4051)	0.8006	73	-1.704 (-2.0007~-1.4073)	0.4759
	>62	131	-1.916 (-2.1923~–1.6397)		132	-1.436 (-1.6631~–1.2089)	
Gender	Male	125	-2.121 (-2.3969~–1.8451)	0.1666	126	-1.646 (-1.8704~-1.4216)	0.4300
	Female	79	-1.477 (-1.864~–1.09)		79	-1.352 (-1.6537~-1.0503)	
Smoking	Smoker	43	-2.382 (-2.8422~–1.9218)	0.2396	43	-0.9060 (-1.273~-0.539)	0.0735
history	non-smoker	161	-1.733 (-1.9919~-1.4741)		162	-1.699 (-1.9036~-1.4944)	
Drinking	Drinker	43	-2.661 (-3.1678~-2.1542)	0.0723	41	-0.9868 (-1.3236~-0.65)	0.1251
history	non-drinker	161	-1.663 (-1.914~-1.412)		164	-1.673 (-1.8812~-1.4648)	
Lymph node metastasis	Yes	94	-1.750 (-2.0767~-1.4233)	0.6153	94	-1.612 (-1.8744~-1.3496)	0.6831
	No	110	-1.978 (-2.2925~-1.6635)		110	-1.464 (-1.7127~-1.2153)	
unknown	0				1	
Distant metastasis	Yes	17	-2.590 (-3.2133~-1.9667)	0.3413	17	-2.013 (-2.7179~-1.3081)	0.4199
	No	187	-1.807 (-2.0471~-1.5669)		188	-1.489 (-1.675~-1.303)	

CRC, colorectal cancer.

### Diagnosis of CRC by 3’tRF-ThrCGTand 3’tRF-mtlleGAT

**Figure 5:**
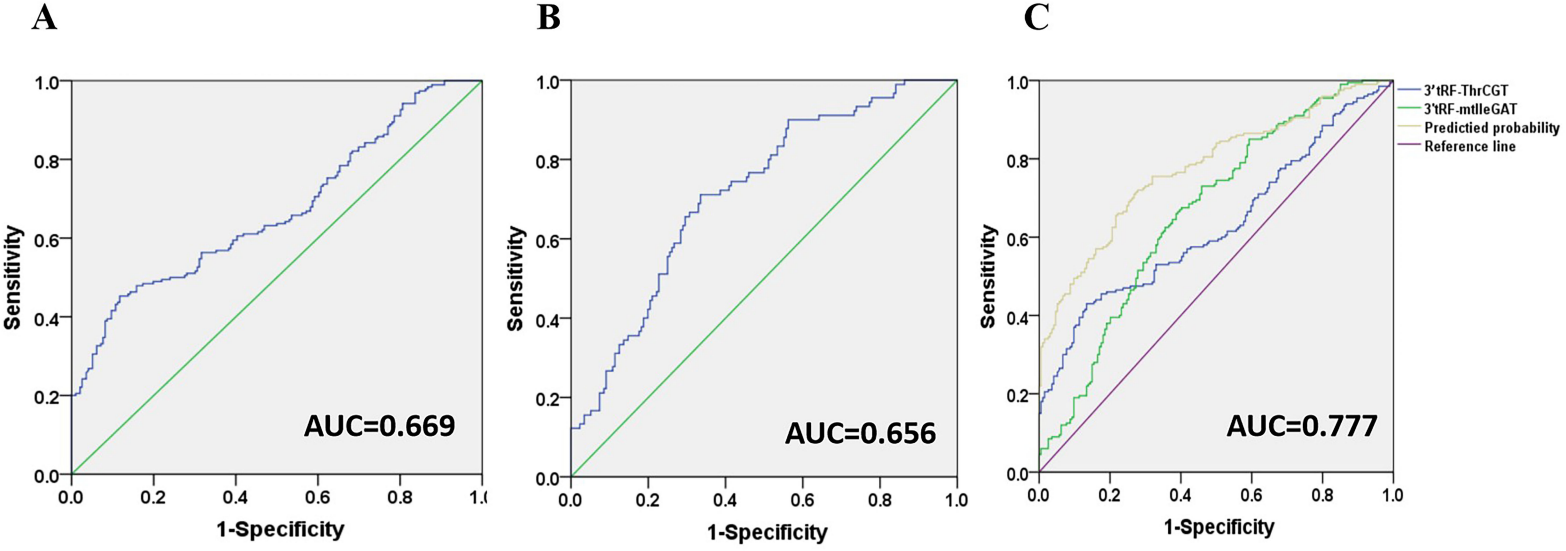
Diagnostic role of serum exosomal 3’tRF-ThrCGT and 3’tRF-mtlleGAT expression levels in CRC patients patients. The AUCs of 3’tRF-ThrCGT **(A)**, 3’tRF-mtlleGAT **(B)**, and both **(C)** in CRC patients relative to healthy donors.

3’tRF-ThrCGT and 3’tRF-mtlleGAT were used as diagnostic CRC indicators, assessing the diagnostic rate using AUC. The 3’tRF-ThrCGT diagnostic rate as a predictor for qPCR screening to diagnose CRC was 0.669 (**Figure 5A**), and the 3’tRF-mtlleGAT diagnostic rate alone was 0.656 (**Figure 5B**). The combined diagnosis of 3’tRF-ThrCGT and 3’tRF-mtlleGAT was 0.777 {95% confidence interval (CI) 0.732–0.822} (**Figure 5C**). The combined diagnosis of CRC by tRF RNAs is more efficient and provides a basis for minimally invasive diagnosis in the clinic.

### 3’tRF-ThrCGTand 3’tRF-mtlleGAT combined tumor markers improve diagnosis of CRC

**Figure 6:**
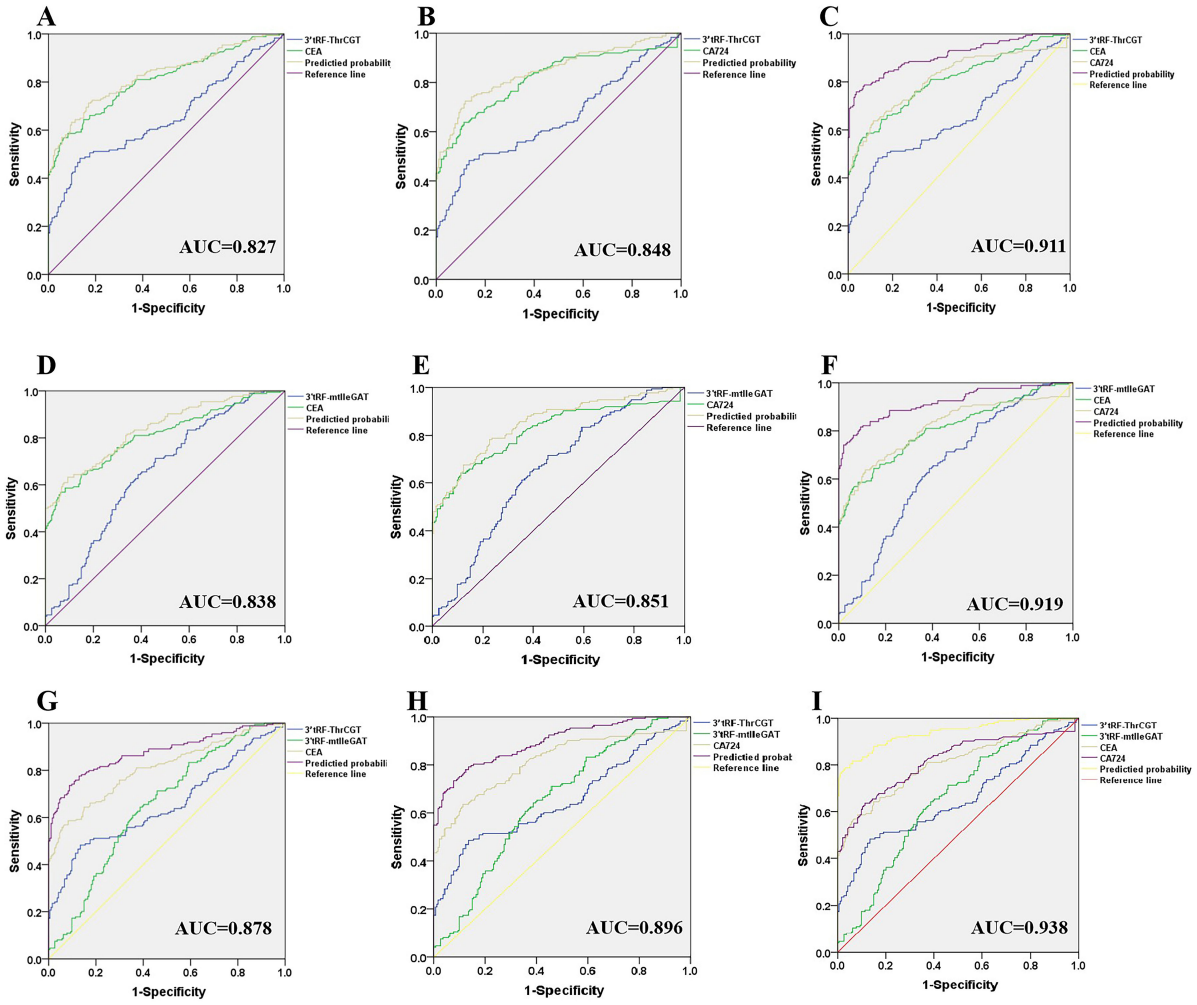
Improved diagnostic capacity of serum exosomes 3’tRF-ThrCGT and 3’tRF-mtlleGAT combined with established tumor markers in CRC patients. The AUCs of 3’tRF-ThrCGT combined with CEA **(A)**, CA724 **(B)**, and both **(C)**. The AUCs of 3’tRF-mtlleGAT combined with CEA **(D)**, CA724 **(E)**, and both **(F)**. The AUCs of 3’tRF-ThrCGT and 3’tRF-mtlleGAT combined with CEA **(G)**, CA724 **(H)**, and both **(I)**.

Currently, tumor-related antigens are mostly utilized as a diagnostic basis for CRC clinical prediction. Carcinoembryonic antigen (CEA) is a tumor-related antigen with low specificity for the prediction of CRC, and it is difficult to determine the organ of origin of the cancer using CEA alone, which has an elevated expression in the process of numerous tumorigeneses. Therefore, it is necessary to combine with other tumor markers for diagnosis. Glycoantigen724 (CA724) is a laboratory marker for detecting gastric cancer and various digestive cancers, which is mainly seen in the gastrointestinal tract and has a higher sensitivity for gastric cancer, ovarian mucinous cystadenocarcinoma, and non-small cell lung cancer, biliary system tumors, CRC, and pancreatic cancer. Combined diagnostic significance, the 3’tRF-ThrCGT combined diagnostic test has significantly improved CRC diagnostic efficiency. 3’tRF-ThrCGT and CEA diagnostic efficiency was 0.827 (**Figure 6A**), 5 ‘tRF-GlyGCC and CA724 diagnostic efficiency was 0.848 (**Figure 6B**), 3’tRF-ThrCGT combined with the two tumor markers diagnostic rate was 0.911{95% CI 0.880–0.942} (**Figure 6C**). 3’tRF-mtlleGAT combined with CEA and CA724 to detect their AUCs, respectively, and it was found that the results of (**Figure 6D**) study showed that 3’tRF-mtlleGAT and CEA diagnostic efficiency was 0.838, 3’tRF-mtlleGAT and CA724 diagnostic efficiency was 0.851 (**Figure 6E**), and 3’tRF-mtlleGAT combined with CEA and CA724 diagnostic rate was 0.919 {95%CI 0.889–0.948} (**Figure 6F**). The diagnostic rate of 3’tRF-ThrCGT and 3’tRF-mtlleGAT combined CEA was 0.878 (**Figure 6G**), and the diagnostic rate of 3’tRF-ThrCGT and 3’tRF-mtlleGAT combined CA724 diagnostic rate was 0.896 (**Figure 6H**), 3’tRF-ThrCGT and 3’ tRF-mtlleGAT combined with both swelling markers diagnostic rate was 0.938 {95% CI 0.913–0.963} (**Figure 6I**).

## Discussion

CRC incidence has shown an increasing trend year after year, and the mortality rate remains high, with many patients found to be in advanced stages since 2020. There are estimated to exceed 1.9 million new CRC cases and 935,000 mortalities in 2020, accompanied by elevated morbidity and mortality rates (27, 28). CRC was diagnosed at an advanced stage, with a low probability of early detection. Therefore, early CRC diagnosis and prevention was crucial (29, 30). Not only has it been significant in improving patient survival, but it also has been crucial in improving the quality of life of the patient’s prognosis (31, 32).

Currently, colonoscopy is widely employed in CRC diagnosis, yet it has many disadvantages, such as higher costs for patients, low acceptance by patients, poor patient tolerance, and difficulty in screening efficiently (28). Generally, as a routine census was difficult to carry out, so simple, minimally invasive diagnostic methods were very necessary. We chose minimally invasive tRF RNA combined with tumor markers as an indicator of screening CRC benefits to avoid the pain of patient colonoscopy and increase patient acceptance (33). In cases where colorectoscopy fails to detect small lesions, we can obtain clearer and more precise results from blood tests; the only disadvantage was that the information we collected from patient specimens lacked the number of early-stage patients. The value of 3’tRF-ThrCGT and 3’tRF-mtlleGAT as an early diagnosis of CRC was not sufficiently clear. Currently, tRF RNAs have been less studied for CRC, which is the innovation in this study (34, 35). In the identification of extracellular vesicles, we did not provide negative control photos. In addition, no apolipoprotein (ApoA/B-I) markers was set up in the Western blot experiment to verify the exclusion of intracystic bodies. In future studies, we will pay attention to setting negative controls in subsequent experiments and conduct validation of apolipoprotein (Apo A/B-I) markers in future experiments.

In this study, we screened that 3’tRF-ThrCGT expression exhibited a significant down-regulation in CRC patients *in vivo*, and 3’tRF-mtlleGAT was significantly overexpressed in patients’ sera, with a high diagnostic efficiency of more than 0.65 for a single tRF RNA, and a much higher diagnostic efficiency for the combination of tumor markers. Glycoprotein CEA constitutes an extensively utilized blood-based molecular marker for CRC and has been shown to be a valuable patient monitoring tool, which is now widely used (36). From a prognostic point of view, detecting the expression of CEA helps determine the occurrence of CRC, especially in patients with metastases. Glycoantigen724 (CA724) is one of the test markers for detecting various cancers of the digestive tract, and it is also a non-specific tumor marker. However, it has a high sensitivity for biliary system tumors, CRC, and pancreatic cancer (37). Our findings revealed a diagnostic effect of 0.938, a high diagnostic efficiency, which provides great support for minimally invasive CRC diagnosis when combined with tumor markers for diagnosis.

The 3’tRF-ThrCGT was statistically related to LNM and tumor distal metastasis, and the study showed a correlation with tumor metastasis. 3’tRF-mtlleGAT was not statistically associated with either metastasis or stage of the tumor. The drawback of this study is that we only collected serum specimens from CRC patients, and there is a lack of data from early-stage patients. Notably, CRC development typically involves a process of transformation from adenoma to cancer (37). CRC incidence is exacerbated by the low detection rate in the evolution of the disease and poor patient compliance, among other important factors (38, 39). Early diagnosis and timely follow-up on cancer are the shortcomings of this study; in the future, our focus will be on improving early cancer diagnosis to provide a more efficient method to treat CRC patients at early stages.

## Conclusion

The 3’tRF-ThrCGT and 3’tRF-mtlleGAT are recently developed diagnostic indicators that have a strong combined diagnostic impact and are highly effective in predicting CRC occurrence. These indicators are expected to become novel predictive variables for clinical use.

## Data Availability

The datasets presented in this study can be found in online repositories. The names of the repository/repositories and accession number(s) can be found in the article/**Supplementary Material**.

## Supplementary material

Supplementary Data Sheet 1 Melt curve of 3’tRF-ThrCGT and 3’tRF-mtlleGAT in HDs and CRC patients. The curve of 3’tRF-ThrCGT **(A)**, 3’tRF-mtlleGAT **(B)**. 1-48 samples are HDs, 49-96 samples are CRC patients.
