# Supplementary figures and images for: Serum extracellular vesicles 3’tRF-ThrCGTand 3’tRF-mtlleGAT combined with tumor markers can serve as minimally invasive diagnostic predictors for colorectal cancer
Source: Front Oncol. 2024 Oct 21;14:1474095. doi: 10.3389/fonc.2024.1474095 (PMC11532659; doi:10.3389/fonc.2024.1474095)

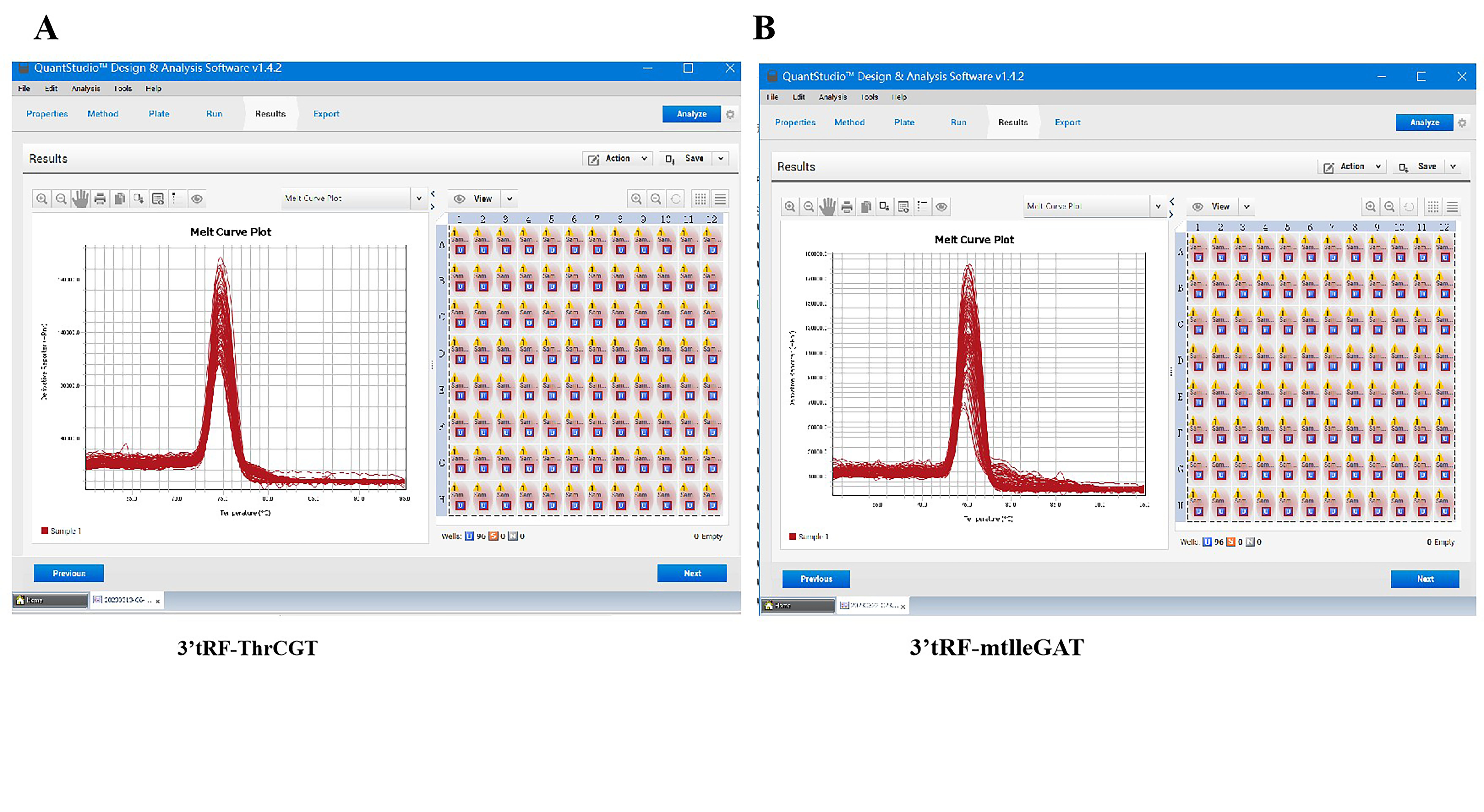

Supplement: Supplementary file 2 [file Image1.jpg]
